# Supplementary material for: Septin crosstalk with microtubules and actin is regulated by a GSK3-dependent phosphoswitch
Source: bioRxiv. 2026 May 9:2026.05.06.723191. Preprint. [Version 1] doi: 10.64898/2026.05.06.723191 (PMC13174562; doi:10.64898/2026.05.06.723191)
Supplement: Supplement 1 [file NIHPP2026.05.06.723191v1-supplement-1.pdf]

bioRxiv preprint doi: https://doi.org/10.64898/2026.05.06.723191; this version posted May 9, 2026. The copyright holder for this preprint (which was not certified by peer review) is the author/funder, who has granted bioRxiv a license to display the preprint in perpetuity. It is made available under aCC-BY 4.0 International license.

Supplementary Figures

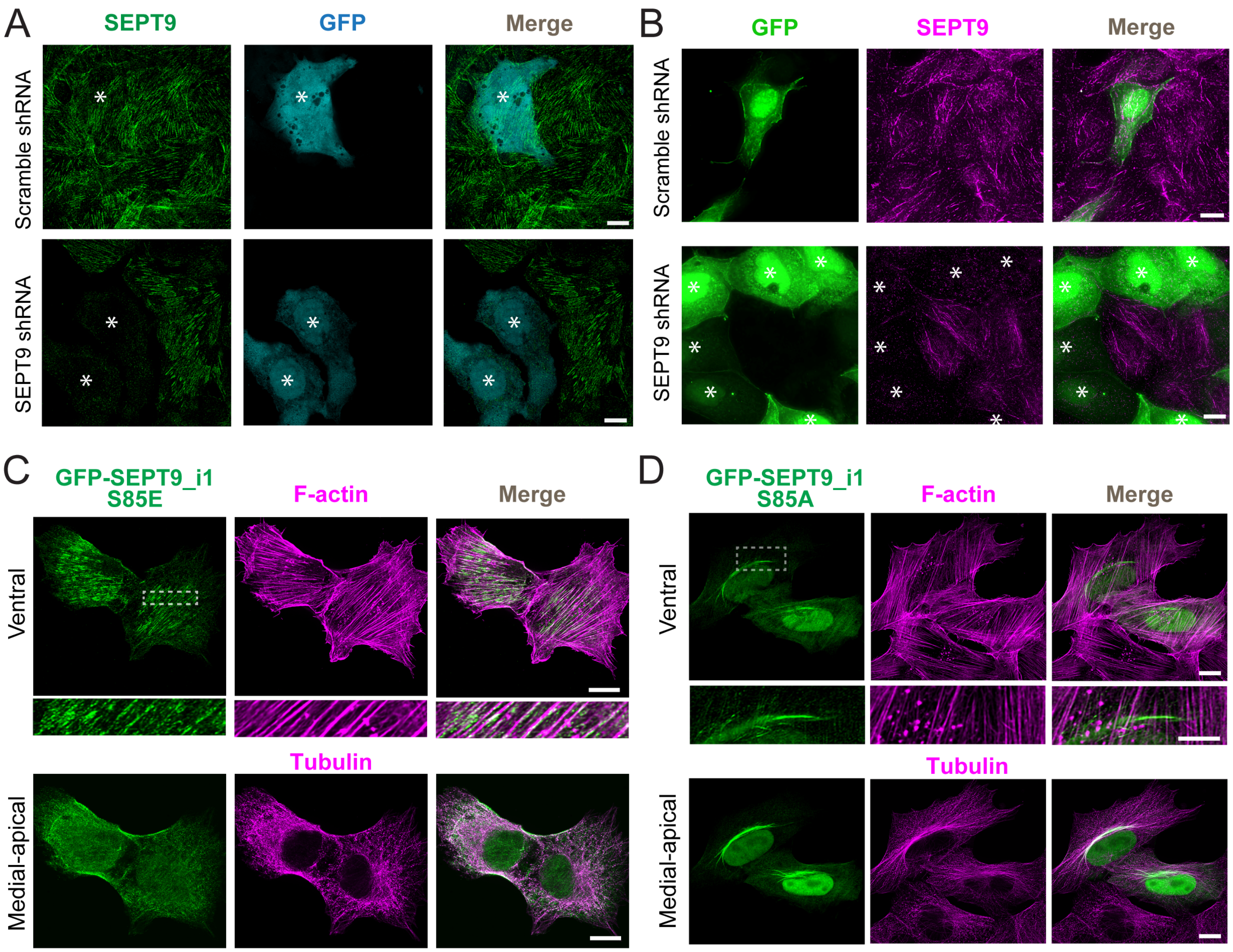

Figure S1. Endogenous SEPT9 depletion, and localization of the SEPT9_i1 mutants S82E and S82A in MDCK cells

(A) Super-resolution SoRa spinning-disk confocal microscopy images show maximum intensity projections of ventral optical sections of U2OS cells transfected with plasmids expressing GFP (cyan) and scramble control (top) or SEPT9 (bottom) shRNAs, and stained for endogenous SEPT9 (green). Asterisks indicate cells transfected with GFP-expressing plasmids. Scale bars, 10 µm.

(B) Denoised and deconvolved wide-field fluorescence microscopy images show maximum intensity projections of medial-apical optical sections of MDCK cells, which were transfected with plasmids

bioRxiv preprint doi: https://doi.org/10.64898/2026.05.06.723191; this version posted May 9, 2026. The copyright holder for this preprint (which was not certified by peer review) is the author/funder, who has granted bioRxiv a license to display the preprint in perpetuity. It is made available under aCC-BY 4.0 International license.

expressing GFP (green) and scramble control or SEPT9 shRNAs, and stained for endogenous SEPT9 (magenta). Asterisks indicate cells transfected with GFP-expressing plasmids. Scale bars, 10 μm.

(C-D) Super-resolution SoRa spinning-disk confocal microscopy images show maximum intensity projections of ventral (top) and medial-apical (bottom) optical sections of U2OS cells transfected with GFP-SEPT9_i1-S85E (green; C) or GFP-SEPT9_i1-S85A (green; D), stained for actin (phalloidin; top in magenta) and α-tubulin (bottom in magenta). Dashed rectangles indicate regions shown at higher magnification. Scale bars, 10 μm.

bioRxiv preprint doi: https://doi.org/10.64898/2026.05.06.723191; this version posted May 9, 2026. The copyright holder for this preprint (which was not certified by peer review) is the author/funder, who has granted bioRxiv a license to display the preprint in perpetuity. It is made available under aCC-BY 4.0 International license.

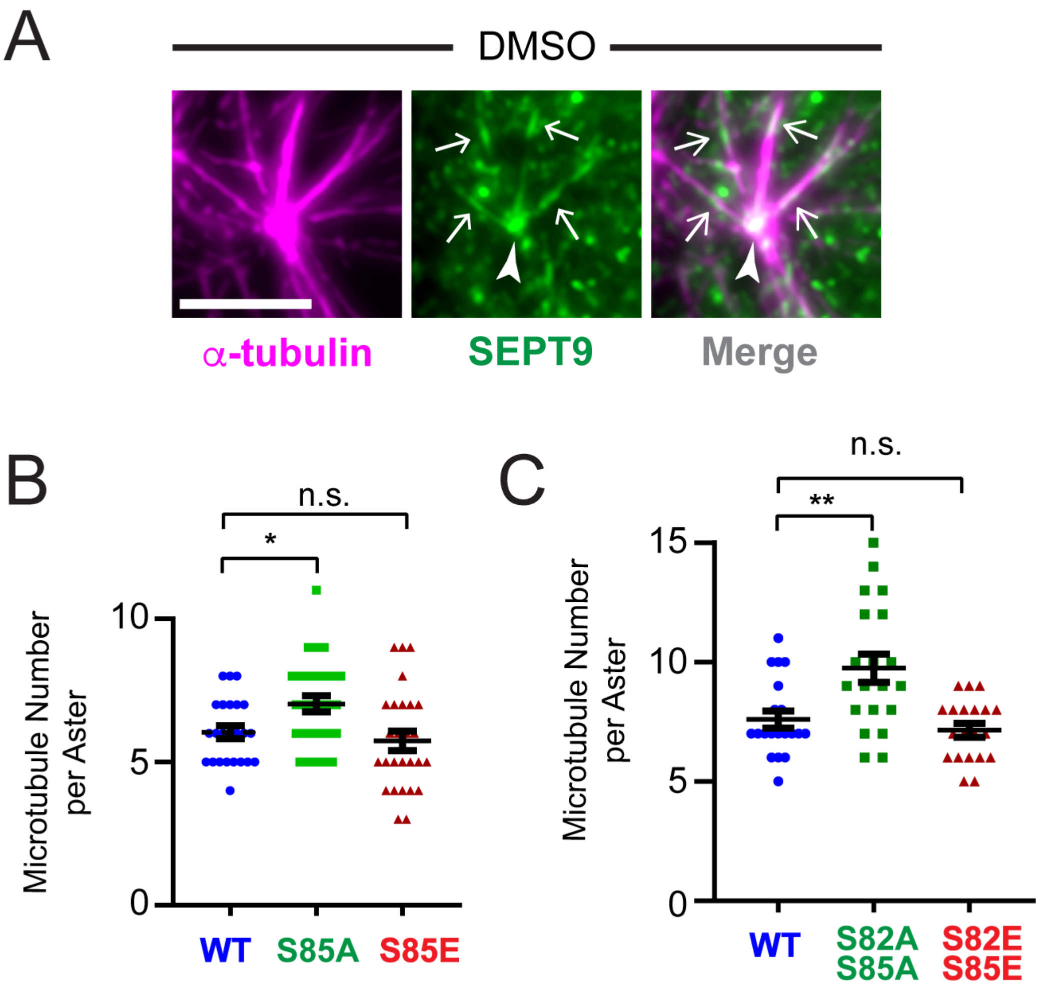

Figure S2. Endogenous SEPT9 localizes to centrosomal microtubules, and phosphonull mutations of SEPT9 at S82 and S85 increase the number of centrosomal microtubules

(A) Denoised and deconvolved wide-field fluorescence microscopy images show maximum intensity projections of medial-apical optical sections of MDCK cells, which were treated with nocodazole (1.6 μM) for 2 h at 37 °C, followed by 40 seconds nocodazole wash-out at 25 °C. Images show endogenous SEPT9 (green) and α-tubulin (magenta). Arrows point to SEPT9 on the lattice of astral microtubules and arrowhead points to SEPT9 at the centrosome. Scale bar, 10 μm.

(B) Quantification of the number (mean ± SEM) of astral microtubules per aster following nocodazole wash-out in MDCK cells transfected with plasmids expressing SEPT9 shRNA and shRNA-resistant wild type GFP-SEPT9_i1, GFP-SEPT9_i1-S85A and GFP-SEPT9_i1-S85E. Pairwise

bioRxiv preprint doi: https://doi.org/10.64898/2026.05.06.723191; this version posted May 9, 2026. The copyright holder for this preprint (which was not certified by peer review) is the author/funder, who has granted bioRxiv a license to display the preprint in perpetuity. It is made available under aCC-BY 4.0 International license.

comparisons between wild-type (*n* = 24 cells), S85A (*n* = 28 cells), and S85E (*n* = 27 cells) mutants were statistically analyzed using a Mann-Whitney U-test. *, $p < 0.01$; n.s., not significant.

(C) Quantification of the number (mean ± SEM) of astral microtubules per aster following nocodazole wash-out in MDCK cells (*n* = 20) transfected with plasmids expressing SEPT9 shRNA and shRNA-resistant wild type GFP-SEPT9_i1, GFP-SEPT9_i1-S82A/S85A and GFP-SEPT9_i1-S82E/S85E. Pairwise comparisons between wild-type, S82A/S85A, and S82E/S85E mutants were statistically analyzed using a Mann-Whitney U-test. **, $p < 0.01$; n.s., not significant.

bioRxiv preprint doi: https://doi.org/10.64898/2026.05.06.723191; this version posted May 9, 2026. The copyright holder for this preprint (which was not certified by peer review) is the author/funder, who has granted bioRxiv a license to display the preprint in perpetuity. It is made available under aCC-BY 4.0 International license.

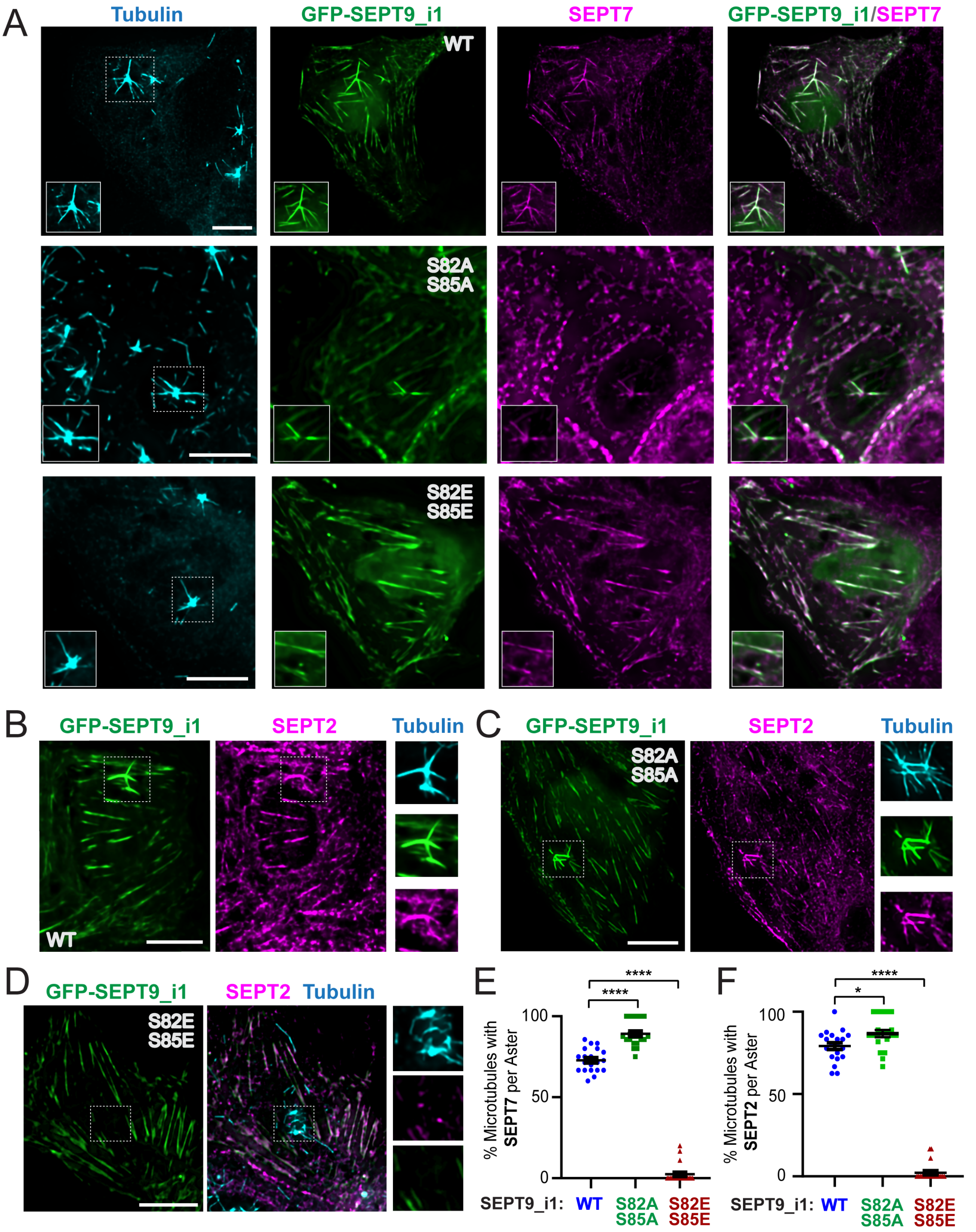

bioRxiv preprint doi: https://doi.org/10.64898/2026.05.06.723191; this version posted May 9, 2026. The copyright holder for this preprint (which was not certified by peer review) is the author/funder, who has granted bioRxiv a license to display the preprint in perpetuity. It is made available under aCC-BY 4.0 International license.

Figure S3. Phosphonull mutations of SEPT9_i1 at S82 and S85 enhance localization of endogenous SEPT7 and SEPT2 to centrosomal microtubules, whereas phosphomimetic mutations have the opposite effect

(A) Denoised and deconvolved wide-field fluorescence microscopy images show maximum intensity projections of medial-apical optical sections of MDCK cells, which were transfected with plasmids expressing SEPT9 shRNA and shRNA-resistant GFP-SEPT9_i1 (wild-type and indicated phosphomutants; green), and stained for endogenous α-tubulin (cyan) and SEPT7 (magenta). Cells were treated for nocodazole (1.6 μM) for 2 h at 37 °C followed by washout for 40 seconds at 25 °C to allow centrosomal microtubule regrowth prior to fixation and staining. Insets show magnified views of regions outlined by dashed rectangles. Scale bars, 10 μm.

(B-D) Denoised and deconvolved wide-field fluorescence microscopy images show maximum intensity projections of medial-apical optical sections of MDCK cells, which were transfected with plasmids expressing SEPT9 shRNA and shRNA-resistant wild type GFP-SEPT9_i1 (B), GFP-SEPT9_i1-S82A/S85A (C) and GFP-SEPT9_i1-S82E/S85E (D), stained for endogenous α-tubulin (blue) and SEPT2 (magenta). Cells were treated for nocodazole (1.6 μM) for 2 h at 37 °C followed by washout for 40 seconds at 25 °C to allow centrosomal microtubule regrowth prior to fixation and staining. Insets show magnified views of regions outlined by dashed rectangles. Scale bars, 10 μm.

(E) Quantification of the percentage (mean ± SEM) of astral microtubules decorated with endogenous SEPT7 per aster following nocodazole wash-out. Pairwise comparisons between wild-type (n = 20 cells) and S82A/S85A (n = 20 cells) or S82E/S85E (n = 20 cells) mutant were statistically analyzed using a Mann-Whitney U-test. ****, $p < 0.0001$.

(F) Quantification of the percentage (mean ± SEM) of astral microtubules decorated with endogenous SEPT2 per aster following nocodazole wash-out. Pairwise comparisons between wild-type (n = 20 cells) and S82A/S85A (n = 20 cells) or S82E/S85E (n = 20 cells) mutant were statistically analyzed using a Mann-Whitney U-test. *, $p < 0.05$; ****, $p < 0.0001$.

bioRxiv preprint doi: https://doi.org/10.64898/2026.05.06.723191; this version posted May 9, 2026. The copyright holder for this preprint (which was not certified by peer review) is the author/funder, who has granted bioRxiv a license to display the preprint in perpetuity. It is made available under aCC-BY 4.0 International license.

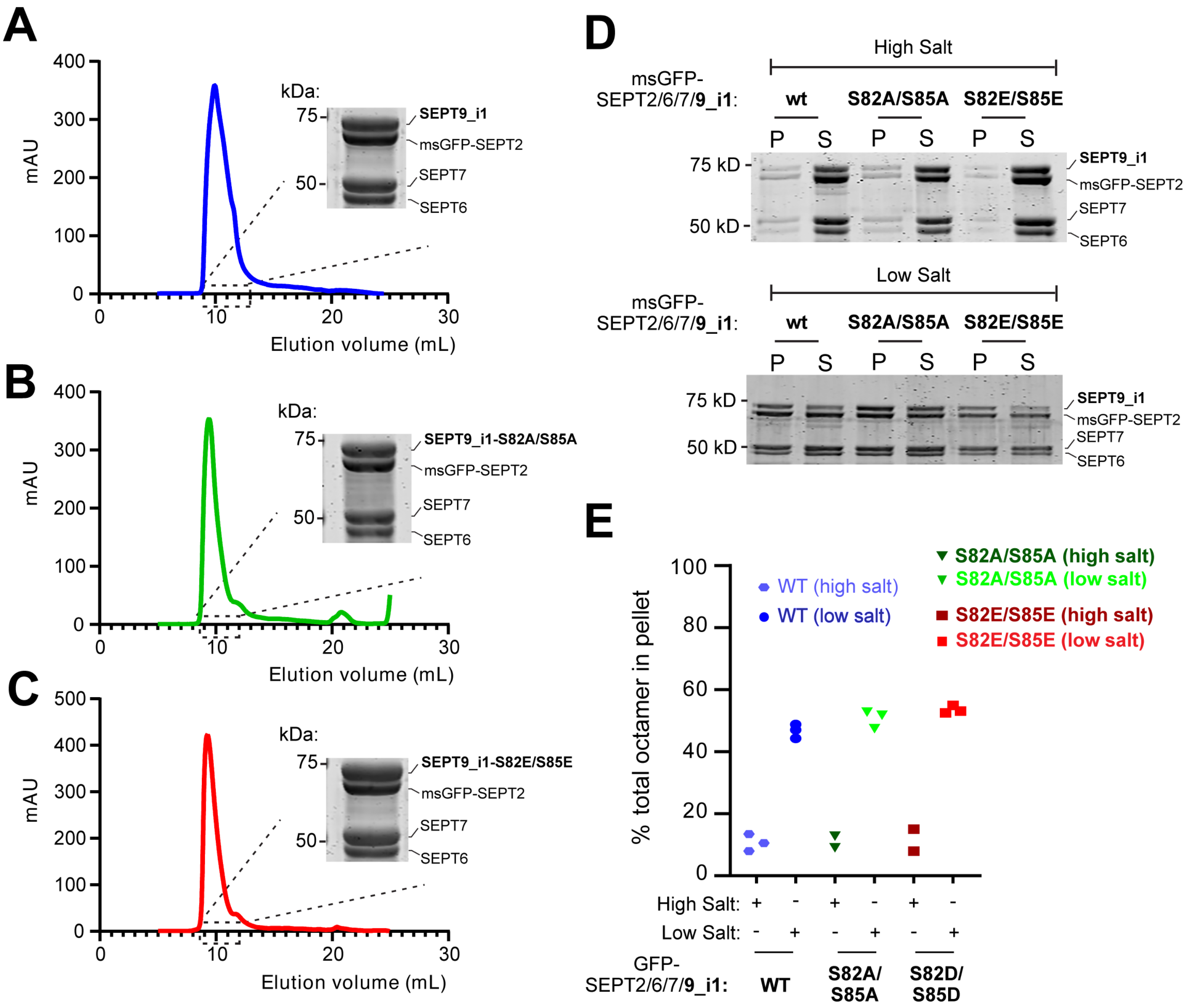

Figure S4. Phosphonull and phosphomimetic mutation of SEPT9_i1 at S82/S85 do not impair assembly into SEPT2/6/7/9 oligomers and polymers.

(A-C) Size-exclusion chromatography elution profiles — absorbance at 280 nm (mAU) as a function of elution volume — of msGFP-tagged SEPT2/6/7/9_i1 complexes following dual tag affinity purification from bacterial lysates that contained Strep-tagged SEPT9_i1 (A), SEPT9_i1-S82A/S85A (B) or SEPT9_i1-S82E/S85E (C) along with SEPT6, SEPT7 and His-/msGFP-tagged SEPT2. Complexes were resolved on a Superdex 200 Increase 10/300 GL column. Coomassie Blue-stained SDS-PAGE gels show the protein composition of fractions pooled from the main elution peaks (dashed rectangles).

bioRxiv preprint doi: https://doi.org/10.64898/2026.05.06.723191; this version posted May 9, 2026. The copyright holder for this preprint (which was not certified by peer review) is the author/funder, who has granted bioRxiv a license to display the preprint in perpetuity. It is made available under aCC-BY 4.0 International license.

(D) Coomassie Blue-stained SDS-PAGE gels of pellet and supernatant fractions from sedimentation assays of SEPT2/6/7/9_i1 complexes containing wild-type, S82A/S85A, or S82E/S85E SEPT9_i1. Complexes were diluted and incubated for 2 h in either high-salt buffer (300 mM KCl; polymerization control) or low-salt buffer (45 mM KCl), which induces assembly into higher-order polymers. Following sedimentation at 39,000 × g, pellets were resuspended in a volume equal to that of the supernatant, and equal volumes of each fraction were resolved by SDS-PAGE.

(E) Quantification of the fraction of total SEPT2/6/7/9_i1 complex protein recovered in the pellet following incubation in high- and low-salt buffer. Data are from three independent experiments in low-salt buffer and two to three independent experiments in high-salt buffer (control).

bioRxiv preprint doi: https://doi.org/10.64898/2026.05.06.723191; this version posted May 9, 2026. The copyright holder for this preprint (which was not certified by peer review) is the author/funder, who has granted bioRxiv a license to display the preprint in perpetuity. It is made available under aCC-BY 4.0 International license.

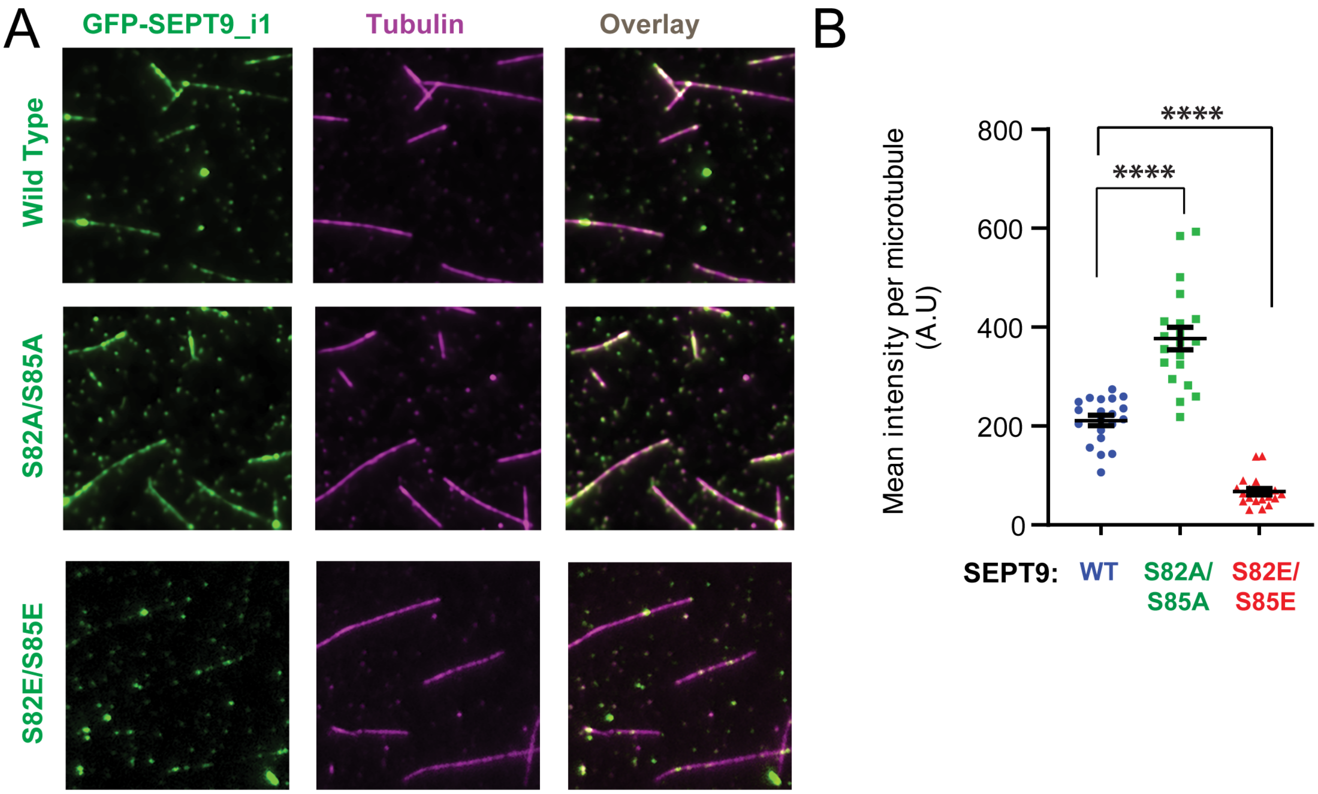

Figure S5. Phosphonull and phosphomimetic mutations of SEPT9_i1 at S82/S85 increase and reduce, respectively, binding to microtubules in vitro

(A) Total internal reflection fluorescence (TIRF) microscopy images of taxol-stabilized microtubules (magenta) following incubation with 3 nM GFP-SEPT9_i1 (green) for wild type, and S82A/S85A, and S82E/S85E mutants. Scale bars, 10 μm.

(B) Quantification of mean GFP fluorescence intensity per microtubule length following incubation with 3 nM GFP-SEPT9_i1 (n = 20; WT, S82A/S85A, S82E/S85E). Pairwise comparisons between WT and S82A/S85A were statistically analyzed using an unpaired Welch's t-test, and between WT and S82E/S85E with a Mann-Whitney U-test. ****, $p < 0.0001$

Plot shows mean ± SEM.

bioRxiv preprint doi: https://doi.org/10.64898/2026.05.06.723191; this version posted May 9, 2026. The copyright holder for this preprint (which was not certified by peer review) is the author/funder, who has granted bioRxiv a license to display the preprint in perpetuity. It is made available under aCC-BY 4.0 International license.

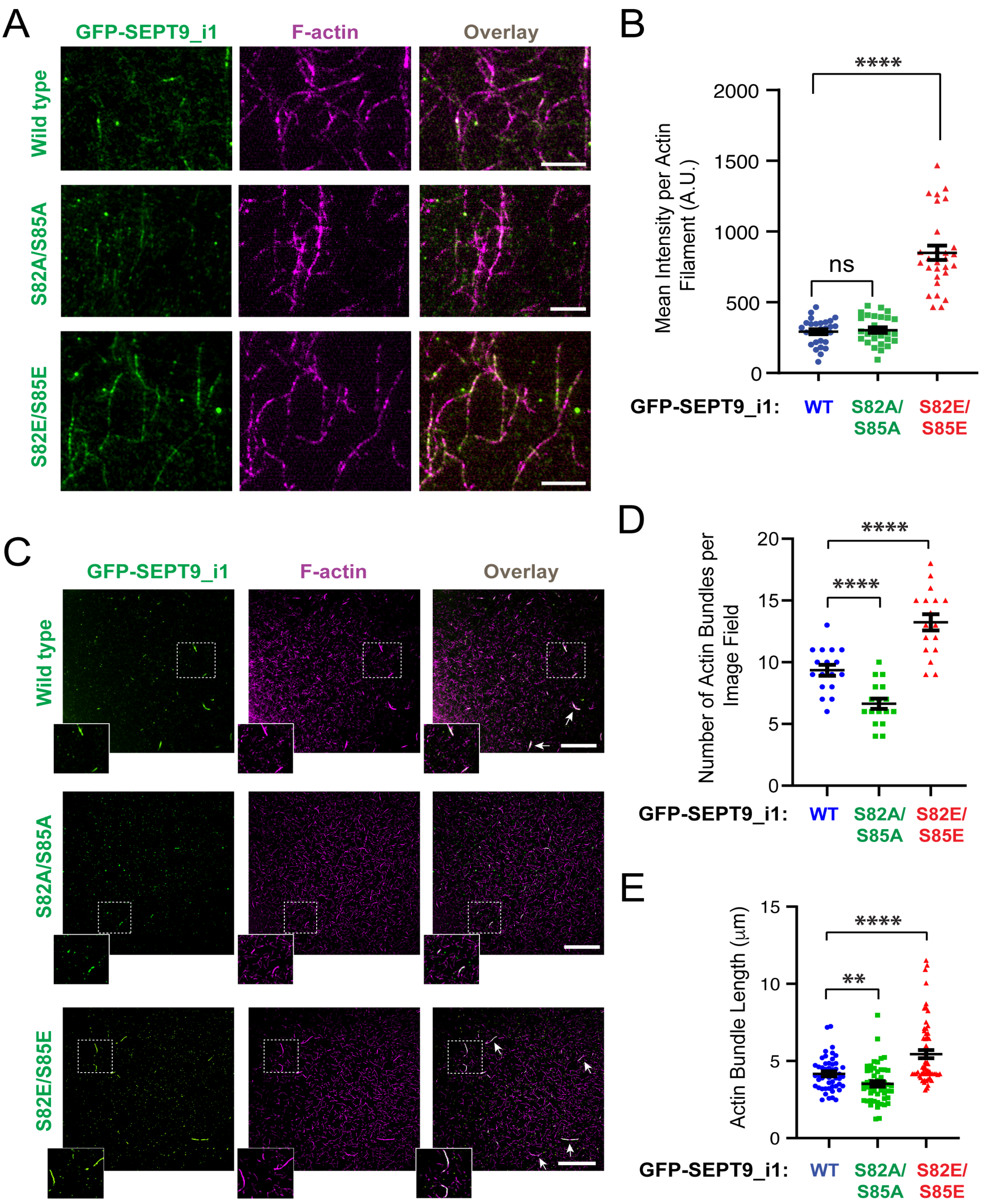

bioRxiv preprint doi: https://doi.org/10.64898/2026.05.06.723191; this version posted May 9, 2026. The copyright holder for this preprint (which was not certified by peer review) is the author/funder, who has granted bioRxiv a license to display the preprint in perpetuity. It is made available under aCC-BY 4.0 International license.

Figure S6. Phosphomimetic mutations of SEPT9_i1 at S82/S85 enhance actin binding and bundling in vitro, and phosphonull mutations decrease actin bundling

(A) Total internal reflection fluorescence (TIRF) microscopy images of phalloidin-stabilized actin filaments (magenta) following incubation with 200 nM GFP-SEPT9_i1 (green) for wild type, and the S82A/S85A and S82E/S85E mutants. Scale bars, 5 μm.

(B) Quantification of mean GFP fluorescence intensity per actin length following incubation with 200 nM GFP-SEPT9_i1 (*n* = 28; WT, S82A/S85A, S82E/S85E). Pairwise comparisons between WT and S82A/S85A was statistically analyzed with a Welch's t-test, and between WT and S82E/S85E with a Mann Whitney U-test. ns, not significant; ****, $p < 0.0001$

(C) Total internal reflection fluorescence (TIRF) microscopy images of phalloidin-stabilized actin filaments (magenta), which were flown into the TIRF chamber following an incubation with 500 nM GFP-SEPT9_i1 (green; wild type, S82A/S85A or S82E/S85E) in solution for 15 minutes at 25 ^{o}C. Insets show at higher magnification regions with actin bundles outlined with dashed rectangles. Arrows point actin bundles. Scale bars, 20 μm.

(D) Quantification of the number (mean ± SEM) of actin bundles per image field (*n* = 17) of equal surface area for GFP-SEPT9_i1, GFP-SEPT9_i1-S82A/S82A and GFP-SEPT9_i1-S82E/S85E. Pairwise comparisons between WT and the mutants were statistically analyzed using an unpaired Welch's t-test. ****, $p < 0.0001$

(E) Quantification of the length (mean ± SEM) of actin bundles for GFP-SEPT9_i1 (*n* = 48), GFP-SEPT9_i1-S82A/S82A (*n* =50) and GFP-SEPT9_i1-S82E/S85E (*n* = 61). Pairwise comparisons between WT and the mutants were statistically analyzed using the Mann-Whitney U-test. **, $p < 0.01$; ****, $p < 0.0001$
